# The effect of Translationally Controlled Tumour Protein (TCTP) on programmed cell death in plants

**DOI:** 10.1186/1471-2229-13-135

**Published:** 2013-09-16

**Authors:** Marion Christine Hoepflinger, Johannes Reitsamer, Anja Maria Geretschlaeger, Norbert Mehlmer, Raimund Tenhaken

**Affiliations:** 1 Department of Cell Biology, Division of Plant Physiology, University of Salzburg, Hellbrunnerstraße 34, 5020 Salzburg, Austria; 2 Department of Biology I, Botany, LMU Munich, 82152 Martinsried, Germany

**Keywords:** TCTP, Programmed cell death, BAX, Tunicamycin, Unfolded protein response, Calcium, Endoplasmic reticulum, Mitochondria

## Abstract

**Background:**

Translationally controlled tumour protein (TCTP), a well known protein of the animal kingdom, was shown to be a Ca^2+^-binding protein with important functions in many different cellular processes (e.g. protection against stress and apoptosis, cell growth, cell cycle progression, and microtubule organization). However, only little is known about TCTP in plants. Transcript and protein levels of plant TCTPs were shown to be altered by various stress conditions (e.g. cold, salt, draught, aluminium, and pathogen infection), and *Arabidopsis thaliana* TCTP (AtTCTP) was described as an important regulator of growth. The aim of this study was to further characterize plant TCTP relating to one of its major functions in animals: the protection against cell death.

**Results:**

We used two different activators of programmed cell death (PCD) in plants: the mammalian pro-apoptotic protein BAX and tunicamycin, an inhibitor of glycosylation and trigger of unfolded protein response (UPR). Over-expression of AtTCTP significantly decreased cell death in tobacco leaf discs in both studies. A ^45^Ca overlay assay showed AtTCTP to be a Ca^2+^-binding protein and localization experiments revealed cytosolic distribution of AtTCTP-GFP in *Arabidopsis* seedlings.

**Conclusions:**

Our study showed cytoprotective effects of plant TCTP for the first time. Furthermore, we showed the ability of AtTCTP to bind to Ca^2+^ and its cytosolic distribution within the cell. If these results are combined, two putative modes of action can be assumed: 1) AtTCTP acts as Ca^2+^ sequester, preventing PCD by reducing cytosolic Ca^2+^ levels as described for animals. 2) AtTCTP could directly or indirectly interact with other cytosolic or membrane-bound proteins of the cell death machinery, thereby inhibiting cell death progression. As no homologous proteins of the anti-apoptotic machinery of animals were found in plants, and functional homologues still remain to be elucidated, future work will provide more insight.

## Background

The translationally controlled tumour protein (TCTP), discovered more than 20 years ago, is ubiquitously expressed throughout all eukaryotes. Sequence analyses revealed a highly conserved protein without homologies to other known proteins (reviewed in [1]). Structure analyses of *S. pombe* TCTP revealed similarities to the Mss4/Dss4 superfamily [2]. Mss4/Dss4 proteins are known to bind to a nucleotide-free (GDP/GTP-free) form of small GTPases, the so called Rab proteins. Up to now, many studies were carried out relating TCTP to multiple cellular processes (e.g. cell growth, cell cycle progression, microtubule organization, ion homeostasis, protection against stress, and apoptosis) including reports of several interacting proteins (e.g. tubulin, Na^+^/K^+^-ATPase, and polo kinase; reviewed in [1]).

Little is known about TCTPs of plants by now. A first study carried out in 1997 associated TCTP to root cap cell division of *Pisum sativum*[3]. Other studies followed and revealed changes in plant TCTP transcript or protein levels in response to different stress conditions like cold, light, salt, water deficit, aluminium, or *Agrobacterium* mediated transformation [4-8]. Furthermore, TCTP levels were shown to decrease in response to pathogen infection [9-11], and a possible involvement in long-distance movement of phloem proteins was assumed [12]. Sequence comparisons of known TCTPs revealed homologous forms in many different plant species [13]. TCTPs of non-plant sequences are usually encoded by a single copy gene, whereas plants have several different *TCTP* cDNAs, pointing to more than one TCTP encoding gene [14]. *Arabidopsis thaliana* owns two TCTP genes, At3g16640 and At5g05540. The gene locus At5g05540 was assumed to be a pseudogene without function, because of a lack of expression in various tissues. Nonetheless, in other plants a possible specialization of different TCTPs was supposed [13]. A first functional characterization of *A. thaliana* TCTP (AtTCTP, At3g16640) was published in 2008 and showed elevated expression levels in mitotically active tissues. TCTP knockout plants revealed a male gametophytic phenotype with impaired pollen tube growth, that prevented creation of homozygous lines. *AtTCTP* silencing resulted in reduced cell size, slower vegetative growth, and altered root development. Besides, AtTCTP was described to affect lateral root formation and auxin homeostasis. Protein alignments showed that of all known protein-protein interaction sites, the GTPase binding site was the only region conserved in animals and plants [13]. An interaction of AtTCTP with different members of small GTPases of plant Rab family (AtRAB4A, AtRAB4b, AtRABF1, and AtRABF2b) was confirmed and a role as positive regulator of mitotic growth was claimed for AtTCTP, presenting a shared function with animals [15]. Another study showed that AtTCTP over-expression is able to confer drought tolerance in *Arabidopsis* by ABA-mediated stomatal closure via TCTP/microtubule interaction, which was enhanced in the presence of calcium. Microtubule binding of TCTP is another shared function of plant, animal and yeast TCTPs [16,17].

In all organisms observed so far TCTP is closely related to calcium. The Ca^2+^-binding ability was originally detected in 1992 by Haghighat and Ruben working on *Trypanosoma brucei*[18]. The expression of mammalian TCTP was shown to be regulated at the transcriptional and post-transcriptional level by calcium, suggesting a role of TCTP in calcium homeostasis [19]. Since then several groups worked on this topic using the radioactive ^45^Ca^2+^ isotope in Ca^2+^ overlay assays to further characterize different TCTPs [20-24]. Mammalian TCTP was assumed to act as a Ca^2+^ sequester, preventing cytosolic Ca^2+^ levels from exceeding apoptosis signalling thresholds, thereby protecting the cell against Ca^2+^-dependent programmed cell death [24].

In this study further information for plant TCTP characterization is provided. We analyzed the Ca^2+^-binding ability of *Arabidopsis thaliana* TCTP and showed the effect of this protein on programmed cell death inducing agents *in vivo*.

## Results

### Effect of AtTCTP on BAX-induced cell death

**Figure 1:**
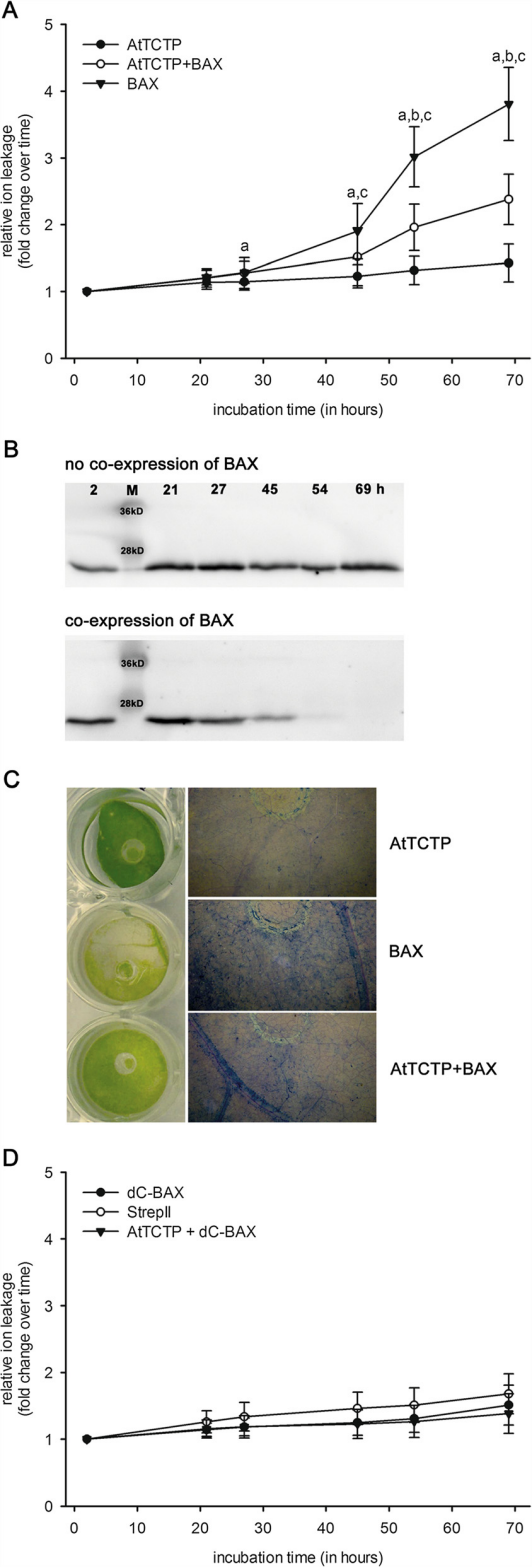
**Effect of TCTP on BAX induced cell death.** Cell death quantification of *N. benthamiana* leaf discs transiently over-expressing *Arabidopsis thaliana TCTP* (*AtTCTP*) and/or *BAX*. **A** shows ion leakage calculated relative to time point T_0_ in response to 2 μM dexamethasone (DEX), which induced expression of BAX. Statistical significances were calculated using analysis of variance (*P <* 0.05) and are indicated as follows: **a** BAX vs. AtTCTP, **b** AtTCTP + BAX vs. AtTCTP, **c** AtTCTP + BAX vs. BAX. **B** shows western blot analyses of leaf discs expressing AtTCTP over time (following time points are shown: 2, 21, 27, 45, 54, and 69 hours of disc floating); AtTCTP is shown without (upper panel) and with (lower panel) co-expression of *BAX*. **C** shows tobacco leaf discs expressing AtTCTP, BAX, or both while floating on 2 μM DEX containing solution for 69 hours (panel on the left side). Panels on the right side show trypan blue staining of dead cells from leaf discs expressing BAX and/or AtTCTP as indicated. **D** shows control experiments: relative ion leakage measurements in response to expression of ΔC-BAX, StrepII-tag, or co-expression of ΔC-BAX + AtTCTP in response to 2 μM DEX. No statistically significant differences were calculated using analysis of variance.

The effect of AtTCTP on the pro-apoptotic effect of BAX, a member of the Bcl-2 family of mouse, was tested in tobacco leaves (Figure 1). Transient expression in *N. benthamiana* was achieved through *Agrobacterium tumefaciens* and relative ion leakage was measured for cell death quantification. Expression of cytotoxic mouse *BAX* was induced by floating transformed tobacco leaf discs on 2 μM dexamethasone. *AtTCTP* was constitutively expressed by the CaMV 35S promoter. 28 hours after induction of BAX expression an increase in ion leakage was observed, that progressively increased until the end of experiment at 69 hours post induction. Co-expression of AtTCTP strongly reduced BAX mediated cell death. Statistical analyses were carried out using analysis of variance (P > 0.05) and are shown in Figure 1A. Expression of AtTCTP over time was observed using Western blot analyses. As shown in Figure 1B, AtTCTP amounts remain stable when expressed without BAX, while BAX co-expression resulted in a slow decrease of AtTCTP over time. As shown in the left panel of Figure 1C, BAX expressing leaf discs revealed clear signs of chlorosis after 69 hours of incubation on 2 μM DEX, whereas AtTCTP over-expressing ones did not. Co-expression of BAX and AtTCTP resulted in slightly chlorotic discs. Trypan blue staining, used to visualize dead cells within leaf discs, provided comparable results (panels on the right side of Figure 1C). The highest amount of dead cells was found in BAX over-expressing tissues, followed by BAX and AtTCTP co-expressing tissues, and AtTCTP over-expressing leaf disc showed almost no dead cells. Control experiments using leaf discs floating on 2 μM dexamethason and expressing either non-apoptotic ΔC-BAX (see [25]), or StrepII-tag without fusion protein, or a combination of ΔC-BAX and AtTCTP are shown in Figure 1D. In contrast to full length BAX, the truncated form (ΔC-BAX) lacks the C-terminal transmembrane region needed for mitochondrial targeting. Therefore, ΔC-BAX is not able to cause cell death (compare [25]). No statistically significant differences were found between controls (analysis of variance).

### Effect of AtTCTP on tunicamycin-induced cell death

**Figure 2:**
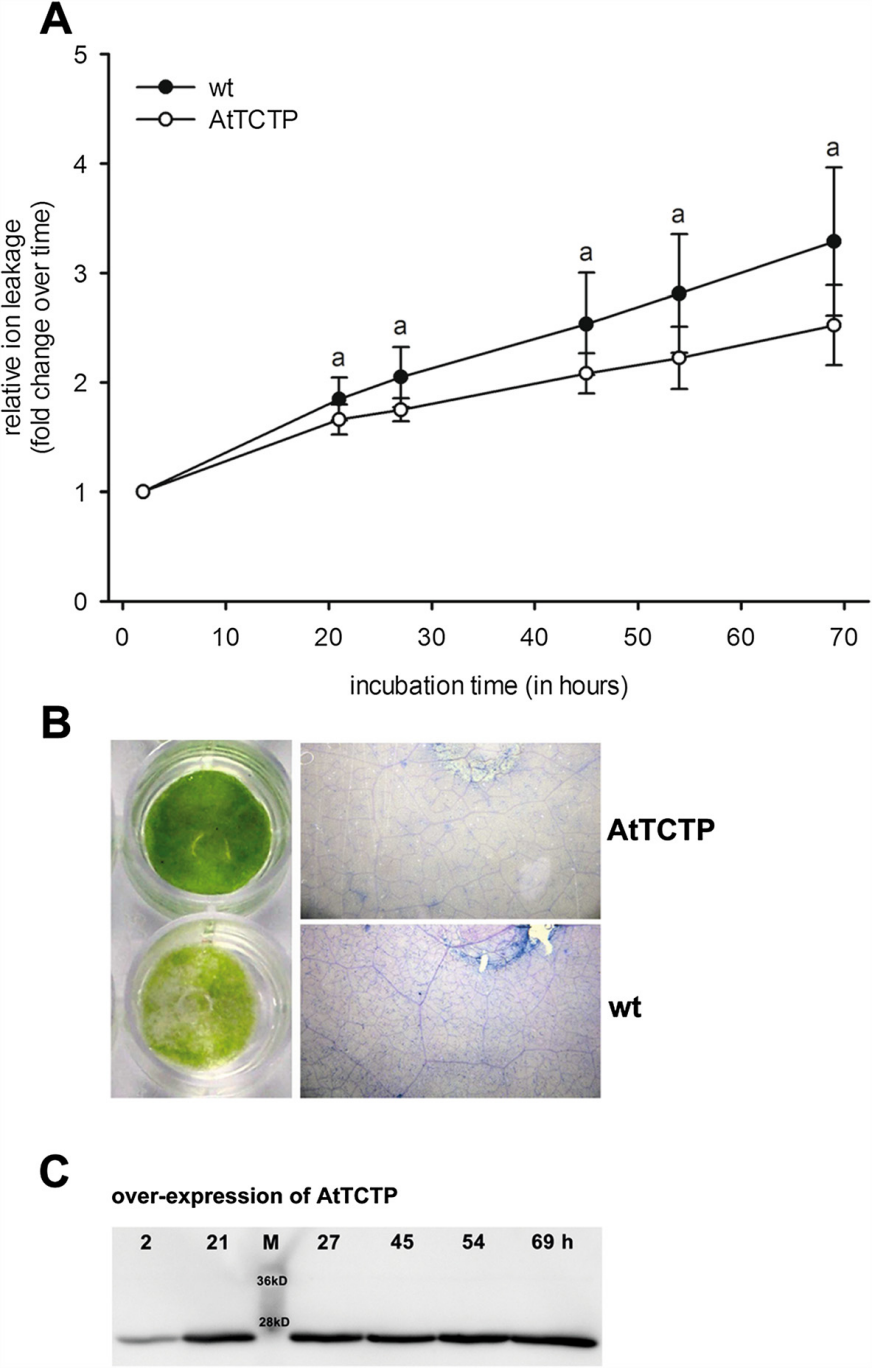
**Effect of AtTCTP on tunicamycin induced cell death.** Cell death quantification of tobacco leaf discs transiently over-expressing *TCTP* of *Arabidopsis thaliana* (AtTCTP). **A** shows ion leakage in response to 2.5 μg · ml^-1^ tunicamycin, calculated relative to time point T_0_. Statistical significances were calculated using analysis of variance (*P <* 0.05; **a** indicates wildtype (wt) vs. AtTCTP over-expression). The panel on the left side of **B** displays tobacco leaf discs floated on 2.5 μg · ml^-1^ tunicamycin for 69 hours, either over-expressing *AtTCTP* or wildtype tobacco (wt). The panel on the right side shows trypan blue staining of dead cells of leaf discs. **C** shows western blot analysis of leaf discs expressing AtTCTP over time (2, 21, 27, 45, 54, and 69 hours of disc floating).

The effect of tunicamycin was probed on AtTCTP over-expressing tobacco leaves. *N. benthamiana* leaf discs transiently expressing *AtTCTP* were examined. Cell death was quantified using relative ion leakage. Tunicamycin is a well known tool for induction of unfolded protein response, which in turn is able to initiate programmed cell death. Figure 2 shows the effect of plant TCTP on tunicamycin induced cell death.

Leaf discs floating on 2.5 μg · ml^-1^ tunicamycin for 69 hours revealed steadily increasing relative ion leakage starting at 21 h after treatment. Statistical analyses were carried out using analysis of variance (P > 0.05) and confirmed the significant reduction of tunicamycin induced ion leakage by AtTCTP protein (see Figure 1A). Figure 2B shows tobacco leaf discs after 69 hours of tunicamycin incubation. AtTCTP expressing discs showed no signs of chlorosis (Figure 2B, left panel), whereas wildtype leaf discs revealed clear signs of chlorosis. Trypan blue staining was used to visualize dead cells within leaf discs. The panel on the right side of Figure 2B shows stained leaf discs floating on 2.5 μg · ml^-1^ tunicamycin for 69 hours. AtTCTP over-expression clearly decreased the amount of trypan blue stained cells when compared to wildtype. Expression of AtTCTP was observed by western blot analyses: AtTCTP expression remained constant during the whole experiment as shown by Western blot detection (see Figure 2C).

### Ca^2+^-binding of AtTCTP

**Figure 3:**
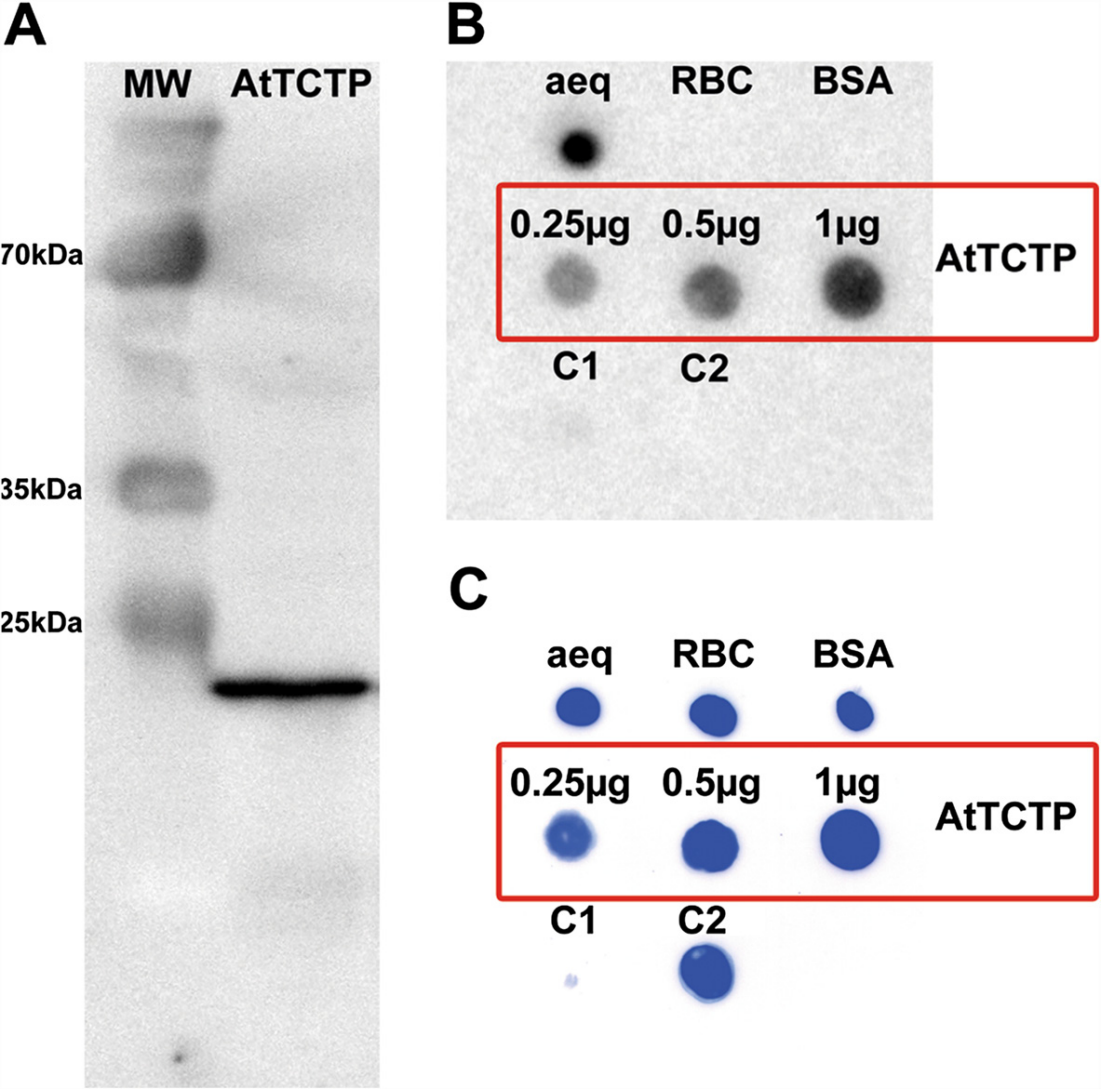
^**45**^**Ca overlay assay of recombinant AtTCTP.** Ca^2+^-binding assay using radiolabelled calcium and recombinant AtTCTP. **A** shows a western blot of recombinant StrepII-tagged AtTCTP (MW = protein marker, AtTCTP = recombinant AtTCTP-StrepII; important marker band sizes are indicated). **B** shows the ^45^Ca overlay assay of AtTCTP, aequorin (aeq), RuBisCo (RBC), bovine serum albumin (BSA), and controls C1 (10 μl purified crude extract without StrepII-tag protein expression) and C2 (non Ca^2+^-binding, StrepII-tagged *A. thaliana* protein kinase). **C** shows Coomassie brilliant blue staining of the PVDF membrane used in 3B. If not indicated differently, 0.25 μg of each protein were spotted for ^45^Ca overlay assay.

For detection of calcium binding properties recombinant StrepII-tagged AtTCTP was expressed and purified from tobacco (*N. benthamiana*, see Figure 3A), followed by a ^45^Ca overlay assay. Figure 3B and C show 0.25 μg of each control protein spotted onto an activated PVDF membrane. Aequorin, with its three EF-hands for Ca^2+^-binding, was used as positive control. Ribulose-1,5-bisphosphat-carboxylase/-oxygenase (RuBisCO) and bovine serum albumin (BSA) were used as negative controls for Ca^2+^-binding proteins. Two controls were used to visualise false positive Ca^2+^-binding: On the one hand the ability of StrepII-tag to bind to calcium was observed and on the other hand possible protein contamination occurring during purification process. Control 1 (C1) represents 10 μl eluate of purified crude tobacco extract without expression of StrepII-tagged proteins. Control 2 (C2) indicates 0.25 μg of a StrepII-tagged, non Ca^2+^-binding protein kinase of *A. thaliana*, demonstrating that StrepII-tag is not binding to Ca^2+^. As expected, none of those negative controls bind to radiolabelled calcium. However, as shown in Figure 3B, AtTCTP binds to ^45^Ca^2+^. Different amounts of AtTCTP-StrepII were tested: 0.25, 0.5, and 1 μg protein were spotted onto PVDF membrane, in order to show increased Ca^2+^-binding. Following ^45^Ca overlay assay, spotted proteins were stained using Coomassie brilliant blue (see Figure 3C).

### Localization of AtTCTP in *Arabidopsis thaliana*

**Figure 4:**
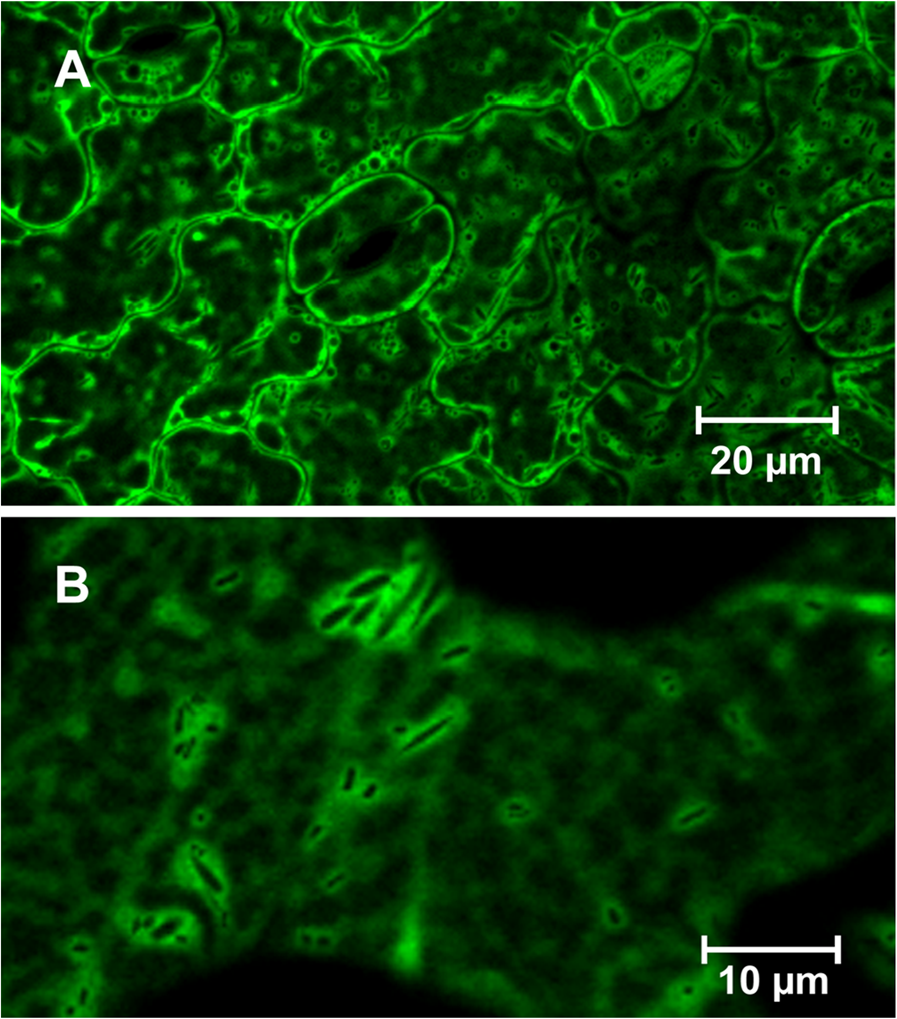
**Localization of AtTCTP.** Distribution of AtTCTP-GFP in seven days old *Arabidopsis thaliana* seedlings. *AtTCTP-GFP* was expressed under the control of a CaMV 35S promoter. **A** and **B** show cytosolic distribution of AtTCTP-GFP in leaf epidermal cells using different magnifications.

Stably transformed, seven to eight days old *A. thaliana* seedlings expressing *AtTCTP::GFP* under the control of a CaMV 35S promoter were used to investigate cellular localization of AtTCTP. Leaf epidermal cells showed cytosolic distribution of AtTCTP-GFP (see Figure 4A, B). The functionality of AtTCTP-GFP was tested in tunicamycin and BAX experiments, revealing the same cytoprotective function as AtTCTP (see Additional file 1: Figure S1). Therefore AtTCTP-GFP was assumed to be functional.

## Discussion

TCTP is well conserved throughout all eukaryotes and described to be involved in many different cellular functions (e.g. cell growth, cell cycle progression, and protection against various stress factors). Although TCTP is well characterized by now, mechanisms of action still need clarification (reviewed in [1]). One of the functions of mammalian TCTP is the protection against apoptosis, which was not described for plants so far. The effect of AtTCTP on programmed cell death in plants was investigated in this study, using two well known inducers of cell death in plants: BAX protein and UPR inducing agent tunicamycin.

Mammalian BAX is a pro-apoptotic member of the Bcl-2 family, involved in PCD initiation by oligomerization and direct association with the outer mitochondrial membrane, hence forming an ion-conducting channel and enabling the passage of macromolecules and other metabolites (e.g. cytochrome c). Furthermore, BAX was shown to induce immediate depletion of endoplasmic reticulum Ca^2+^ pools by integrating into ER membranes, leading to Ca^2+^ efflux, mitochondrial sensitization, and inducing apoptosis [26]. In animals the pro-apoptotic activity of BAX is blocked by anti-apoptotic Bcl-2 and Bcl-X proteins [27]. Although those proteins are key players in ER- and mitochondrion-mediated PCD in animals, no homologous proteins were detectable in plants. Nonetheless, over-expression of BAX is a well-established tool for PCD induction *in planta*[25], and a functional BAX suppressor protein was found in *Arabidopsis thaliana*: AtBI-1 (*A. thaliana* BAX inhibitor protein 1 [25,28]). Several other homologous proteins of AtBI-1 were found in *A. thaliana* and categorized into a new gene family, called ABR (AtBI-related proteins, see [29]). All those studies point to the possibility of a BAX-mediated PCD pathway similar in animals and plants.

As TCTP was shown to act in an anti-apoptotic way in different organisms, we tested the effect of plant TCTP on BAX-induced cell death in tobacco. As shown in Figure 1, over-expression of AtTCTP clearly suppressed the pro-apoptotic effect of BAX indicated by reduced ion leakage levels, a reduced extent of chlorosis, and a diminished amount of dead cells. The decreasing amount of AtTCTP in leaf discs co-expressing AtTCTP and BAX is most likely explained by the increasing amount of dead and dying cells that are not able to express AtTCTP any more (compare Figure 1B,C). A putative mode of action of BAX and TCTP was supposed in mammals: TCTP was described to antagonize the pro-apoptotic effect of BAX without direct interaction. Homodimeric TCTP was assumed to bind to Mcl1 and Bcl-xL, both anti-apoptotic members of the Bcl-2 family. This complex anchors at the outer mitochondrial membrane and separates BAX homodimers, thereby turning off its pro-apoptotic effect [30]. As our results point to an anti-apoptotic effect of AtTCTP on mammalian BAX-induced PCD *in planta*, and as plant proteins have been discovered to have comparable functions to mammalian Bcl-2 family proteins, it could be assumed that AtTCTP acts in a similar way as mammalian TCTP. Unfortunately, there are no homologous proteins of Mcl1 and Bcl-xL in plants, and functional homologues still remain to be elucidated.

In order to further investigate the effect of AtTCTP in plant cell death, we probed another cytosolic Ca^2+^ elevating and PCD-inducing agent, called tunicamycin. Tunicamycin consists of a mixture of homologous nucleoside antibiotics and inhibits protein glycosylation in the ER, leading to an accumulation of inaccurately folded proteins [31]. If this protein misfolding is excessive or persistent, it is able to induce the so called unfolded protein response (UPR), a cellular stress response conserved throughout all eukaryotes, which is destined to re-establish normal cellular functions. UPR is thought to stop protein synthesis and activates signal pathways leading to an increase of polypeptide folding proteins (e.g. binding protein (BiP), protein disulfide isomerase (PDI), calreticulin, and calnexin). If UPR is not able to restore cellular functions within a certain period of time, PCD is initiated [32,33]. In mammals the interplay of tunicamycin and TCTP was observed at mRNA and protein levels using three different cytosolic Ca^2+^ elevating agents: tunicamycin, thapsigargin, and ionophore A23187. TCTP mRNA levels were not influenced by tunicamycin, whereas thapsigargin and ionophore A23187 induced an up-regulation. Similar results were obtained for protein levels: generally TCTP protein levels were down-regulated in response to cytosolic Ca^2+^ elevation, but not in response to tunicamycin. Furthermore, the cell death inducing effect of tunicamycin was inhibited by TCTP over-expression in mammalian cell culture. According to these results it was assumed that direct Ca^2+^ depletion – as provoked by thapsigargin and A23187 – selectively activates signalling pathways other than UPR [19,34]. As tunicamycin is neither altering mRNA nor protein levels of TCTP, the effect of TCTP on tunicamycin induced stress can be assumed to mark the direct effect of existing TCTP levels in the cell. In our study, tunicamycin increased cell death significantly in wildtype when compared to AtTCTP over-expressing tobacco tissues, as shown in Figure 2. Moreover, leaf discs over-expressing AtTCTP showed no chlorosis and a smaller amount of dead cells by trypan blue staining after 69 hours floating on tunicamycin, visualizing the anti-apoptotic effect of AtTCTP.

As both stressors used in this study (BAX, tunicamycin) are known to elevate intracellular Ca^2+^ levels, and TCTP is described as a Ca^2+^-binding protein in non-plant organisms (e.g. [20-24]), we tested the ability of plant TCTP to bind to calcium. Using a ^45^Ca overlay assay, we showed that AtTCTP is a Ca^2+^-binding protein (see Figure 3). The exact mode of action of TCTP binding to calcium still remains to be elucidated. While aequorin, which was used as a positive control in this study, contains EF-hands as typical Ca^2+^-binding motifs, TCTP does not belong to any known family of Ca^2+^-binding proteins [21]. Even within the Ca^2+^-binding region of human TCTP, which consists of a stretch of 30 amino acids, only three out of 6 amino acid residues are conserved between human and plant TCTPs. If this 30 amino acid stretch of different TCTP sequences is compared for human, yeast, *Drosophila melanogaster*, and *A. thaliana*, only a single glutamic acid residue is conserved, which alone is unlikely to enable strong Ca^2+^-binding. This suggests that different organisms have developed multiple ways to establish Ca^2+^-binding of their TCTPs during evolution.

In order to investigate the cellular localization of AtTCTP *in vivo*, we used stably transformed *A. thaliana* seedlings expressing AtTCTP-GFP. As shown in Figure 4, AtTCTP-GFP revealed cytosolic distribution. Mammalian TCTP was predominantly found in cytosol and nucleus *in vivo*, although it was described as a protein functioning in mitochondria [35]. In yeast, the mammalian TCTP ortholog Mmi1p was shown to translocate from cytosol to mitochondria in response to oxidative stress, but unlike other TCTPs Mmi1p was described to exhibit pro-apoptotic effects [17]. As TCTP is hydrophilic and contains no hydrophobic transmembrane domain or any other organelle localization signal, it is most likely that TCTP exhibits its anti-apoptotic function either by quenching cytosolic Ca^2+^, thereby inhibiting PCD signalling, or by interacting with other proteins located at/in the membrane of ER and mitochondria.

## Conclusions

This study showed programmed cell death inhibiting effects of a plant translationally controlled tumour protein for the first time. *Arabidopsis thaliana* TCTP significantly diminished cell death induced by tunicamycin and expression of BAX protein, respectively. Furthermore, we showed AtTCTP to be a Ca^2+^-binding protein located in the cytosol.

If a PCD signal is passed within a cell, Ca^2+^ is released from internal stores (e.g. ER and mitochondria). As AtTCTP is a potent Ca^2+^-binding protein and located within the cytosol, two modes of action can be assumed: on the one hand AtTCTP could be thought to act as a cytosolic Ca^2+^ sequester, preventing further PCD signalling by reducing cytosolic Ca^2+^ levels. A similar mode of action was suggested for mammalian TCTP [24]. The scavenger theory was partially supported by the work of Bommer and colleagues, relating TCTP to cytoprotective functions under Ca^2+^-induced stress [34]. On the other hand TCTP was described as direct or indirect interaction partner of different proteins of the anti-apoptotic machinery of animals: BAX, Bcl-xL, and Mcl-1 [30,36,37]. In general, the same could be supposed for plants, but up to now no homologous forms of interacting proteins were detected in plants and functional homologues remain to be elucidated as well.

## Methods

### Vector construction

In order to generate AtTCTP (At3g16640; GenBank: AEE75847.1) and AtTCTP-GFP expressing vectors *AtTCTP* and *AtTCTP::GFP* were ligated into Gateway compatible vector pXCSG containing the constitutive 35S promoter of cauliflower mosaic virus and a C-terminal StrepII-tag [38], resulting in pXCSG-*AtTCTP* and pXCSG-*AtTCTP::GFP*. Therefore, total RNA was extracted from *Arabidopsis thaliana* using TRI-reagent according to manufacturers’ instructions (Sigma). Residual genomic DNA was removed by RNase-free DNase treatment (Fermentas, #EN0521) and first-strand cDNA was synthesized using 1 μg of total RNA, an anchored oligo(d)T primer-mix, and M-MuLV Reverse Transcriptase (Fermentas, RevertAid #EP0441) according to the suppliers protocol. The following primers were used in a 2-step protocol to design *attB-AtTCTP* and *attB-AtTCTP::GFP* using Phusion High-Fidelity DNA polymerase (Thermo Scientific, #F530S): attB1-adapter: 5′-GGGGACAAGTTTGTACAAAAAAGCAGGCTTC-3′; attB2-adapter: 5′-GGGGACCACTTTGTACAAGAAAGCTGGGT-3′; attB1-AtTCTP: 5′-AAAAAGCAGGCTTCATGTTGGTGTACCAAGATCT-3′; attB2-AtTCTP: 5′-GAAAGCTGGGTCGCACTTGACCTCCTTCA-3′; attB2-GFP: 5′-GAAAGCTGGGTGGCCTCGTCCATCTG-3′. Gateway cloning was performed using Gateway BP and LR Clonase® Mix II from Invitrogen.

Dr. Maki Kawai-Yamada kindly provided the dexamethason (DEX) inducible expression vectors for BAX (GenBank: NP_031553.1; vector: pTA7002-*Bax*) and ΔCBax (pTA7002-*ΔCBax*) described in [25].

### Cultivation of plants

Thale cress (*Arabidopsis thaliana*, ecotype Columbia; NASC, #N60000) was grown on standard fertilized soil (type ED73) after at least 20 hours of stratification in a growth chamber with 16 hours of light and 8 hours darkness. The temperature was set to 23°C during light and 20°C during the dark phase, and humidity was set to 60%. Tobacco (*Nicotiana benthamiana*) seeds were grown the same way but without stratification.

*A. thaliana* seeds were surface sterilized (2.5% sodium hypochlorite) and grown on MS agar plates (0.5× MS strength, 1% phytagel, 0.5% sucrose) in a growth chamber with a 16 hours light/8 hours dark rhythm at 20°C. In order to investigate tunicamycin induced stress, seedlings placed on MS-plates were sprayed with 2.5 μg · ml^-1^ tunicamycin.

### Transient transfection of tobacco leaves

Transient transfection of tobacco leaves (*Nicotiana benthamiana*) was performed as follows: *A. tumefaciens* (strain GV3101) were inoculated in YEB medium supplemented with appropriate antibiotics (for pXCSG vector: 50 μg · ml^-1^ kanamycin, 25 μg · ml^-1^ gentamycin, and 100 μg · ml^-1^ carbenicillin; for P19 repressor 50 μg · ml^-1^ kanamycin and 25 μg · ml^-1^ gentamycin; and for pTA7002 vector: 50 μg · ml^-1^ kanamycin) and grown at 28°C to late exponential phase under continuous shaking. Bacteria were harvested by centrifugation (6,000×g, 5 min, RT); the pellet was resuspended in 5 ml infiltration buffer (10 mM MES-KOH pH 5.6, 10 mM MgCl_2_, and 150 μM acetosyringone). Concentration of bacterial suspensions was photometrically determined (OD_600_); suspensions were mixed to adjust final concentrations: OD_600_ = 0.5 for *Agrobacterium* carrying AtTCTP, AtTCTP-GFP, BAX, ΔCBAX, or StrepII expressing plasmids; OD_600_ = 0.25 for P19 repressor. These suspensions were incubated for two hours at room temperature under continuous shaking, followed by infiltration into four weeks old tobacco leaves using a 1 ml syringe without needle. For further treatment infiltrated leaf areas were marked with a pen in order to precisely recognize the infiltrated parts. For protein expression plants were placed in a growth chamber (8 hours light, 16 hours dark; temperature and humidity as described above) [38,39].

### Cell death quantification: relative ion leakage and trypan blue staining

In order to quantify cell death in tobacco, we used relative ion leakage measurements as described in [40,41]. In brief, tobacco was transiently transfected as described above. Plants were allowed to express proteins for 24 hours prior to leaf disc punching using a leaf puncher of 8 mm in diameter. Leaf disc fresh weight (in grams) was determined and each disc was immediately floated on 1 ml of distilled water containing either dexamethason or tunicamycin (both solved in DMSO) in a well of a 24-well tissue culture plate. DMSO without any additions was used for control conditions. A top cover prevented evaporation and plates were placed under a luminous field (16 hours light, 8 hours darkness; 23°C) during incubation. An electrical conductivity meter (Horiba B-173, Japan) was used to measure the conductivity of the floating solution (in μS · cm^-1^) at different time points: 2, 4, 6, 21, 27, 45, 54, and 69 hours after leaf disc punching and floating. As wounding due to disc punching resulted in a slow but measurable increase in conductivity within the first two hours of floating, the conductivity after two hours was taken as T_0_-value. Relative ion leakage was calculated using the equation described in [40]. Resulting relative ion leakage values were compared and statistically significant differences were evaluated using analysis of variance (ANOVA).

Trypan blue staining was performed according to the protocol of [42]. After staining the discs were washed in glycerol (50%, v/v), placed in a transparent envelope and photographed using a Canon PowerShot S40 camera and a Leica MZFLIII binocular.

### Western blot analyses

For Western blot analyses frozen leaf discs were homogenized using a ball mill (30 Hz) and supplemented with extraction buffer (100 mM HEPES (pH 8.0), 100 mM NaCl, 5 mM EDTA (pH 8.0), 5 mM DTT, 0.5% Triton X-100, 100 μg · ml^-1^ avidin). The extract was shaken by hand and vortexed vigorously while thawing on ice. After thawing the extract was supplemented with one volume of SDS-sample buffer, incubated on 95°C for 5 min and placed on ice until further use. Proteins were separated using SDS-PAGE and blotted onto a PVDF membrane. Nonspecific protein binding sites were blocked using TBST-BSA (1%, w/v) for 60 min, followed by four wash steps (5 min each) in TBST. The membrane was incubated for 10 min in TBST supplemented with 2 μg · μl^-1^ avidin. Strep Tactin alkaline phosphatase conjugate (IBA BioTAGnology, Göttingen) was added in a dilution of 1:50,000 directly to avidin containing TBST and the membrane was incubated for another 60 min. Four wash steps were used to remove unbound conjugate (each time for 5 min). Signal detection was achieved using CDP-Star® reagent (New England Biolabs, Frankfurt, Germany) according to the manufacturers’ instructions and chemiluminescence was detected using a LAS-3000 mini (Fujifilm, Düsseldorf, Germany).

### Purification of recombinant AtTCTP

After transient transfection tobacco leaves (*N. benthamiana*) were allowed to express AtTCTP-StrepII for three days. Marked areas were cut from leaves and ground in liquid nitrogen using mortar and pistil. Extraction buffer was added (100 mM HEPES (pH 8.0), 100 mM NaCl, 5 mM EDTA (pH 8.0), 5 mM DTT, 0.5% Triton X-100, 100 μg · ml^-1^ avidin) and the extract was mixed several times vigorously while thawing on ice. After thawing the extract was incubated for another 10 minutes on ice, followed by two centrifugation steps (15,000×g, 10 min, 4°C) in order to pellet cell debris. The residual supernatant was filtered through two layers of miracloth (Calbiochem/Merck, Darmstadt, Germany) to remove any residual particles. This crude extract was used for Strep-Tactin batch purification using Strep-Tactin® Macro Prep 50% suspension (IBA Göttingen, Germany). All purification steps were carried out at 4°C. Prior to crude extract loading, resin was equilibrated for 20 min on a rotation wheel in at least 2 column volumes extraction buffer. Equilibrated resin was centrifuged (500×g, 2 min) and supernatant was removed prior to loading of the crude extract, followed by incubation for 10 min on a rotation wheel. The sample was centrifuged (500×g, 2 min), the supernatant was removed again, and the resin was washed with 10 column volumes of wash buffer for 4 to 5 times, each time 10 min on a rotation wheel (wash buffer: 100 mM HEPES (pH 8.0), 100 mM NaCl, 0.5 mM EDTA (pH 8.0), 2 mM DTT, 0.005% Trition X-100). Strep-tagged AtTCTP was eluted by incubating Strep-Tactin® resin two times (each time for 5 min) in 3.75 column volumes of elution buffer on an Eppendorf Thermomixer (1,000 rpm) at room temperature (elution buffer: wash buffer supplemented with 10 mM desthiobiotin). Eluted volumes were pooled and 20% glycerol was added for storage (−20°C).

### ^45^Ca overlay assay

The Ca^2+^-binding ability of AtTCTP was detected using ^45^Ca autoradiography described in [43]. Aequorin was used as a positive control; Ribulose-1,5-bisphosphat-carboxylase/-oxygenase (RuBisCO, purified from *Brassica rapa* subsp. *pekinensis*) and bovine serum albumin (BSA) were used as negative controls of Ca^2+^-binding proteins. For examination of StrepII-tag Ca^2+^-binding abilities a StrepII-tagged, non Ca^2+^-binding protein kinase of *A. thaliana* was expressed and purified under conditions described for AtTCTP-StrepII. Moreover, in order to identify any background protein contamination, crude tobacco leaf extract was purified and tested. Of each protein 0.25 μg were spotted onto an activated PVDF membrane (if not indicated differently in Figure 3), corresponding to the approximate amounts of substance: aequorin = 12 pmol, RuBisCo = 14 pmol, BSA = 3.8 pmol, StrepII-tagged protein kinase = 3.8 pmol, and AtTCTP-StrepII = 12 pmol (for 0.25 μg).

## Abbreviations

TCTP: Translationally controlled tumour protein; ER: Endoplasmic reticulum; PCD: Programmed cell death; DEX: Dexamethasone; UPR: Unfolded protein response; DIC: Differential interference contrast microscopy.

## Competing interests

The authors declare that they have no competing interests.

## Authors’ contributions

MCH carried out the experimental studies (except ^45^Ca overlay assay) including statistical analysis, participated in vector design, microscopic studies, and drafted the manuscript. JR participated in molecular cloning, microscopic studies and created the protein over-expressing *A. thaliana* plant line. AMG participated in experimental studies. NM carried out the ^45^Ca overlay assay. RT and MCH planned the study. RT participated in design and coordination and helped drafting the manuscript. All authors read and approved the final manuscript.

## Supplementary Material

Additional file 1: Figure S1 Effect of AtTCTP-GFP on cell death induced by BAX or tunicamycin. Description of data: Confirmation of AtTCTP-GFP functionality in tunicamycin and BAX experiments. Cell death quantification of tobacco leaf discs transiently over-expressing either AtTCTP or AtTCTP-GFP. **A** and **B** show ion leakage calculated relative to time point T_0_ in response to either expression of BAX induced by 2 μM dexamethasone (DEX) (**A**), or in response to 2.5 μg · ml^-1^ tunicamycin (**B**). Statistical significances were calculated using analysis of variance (*P <* 0.05) and are indicated as follows: for **A**: **a** indicates BAX over-expression (BAX) vs. over-expression of both BAX and AtTCTP (AtTCTP + BAX), **b** indicates BAX over-expression (BAX) vs. over-expression of both BAX and AtTCTP-GFP (AtTCTP-GFP + BAX); for **B: a** indicates wildtype (wt) vs. AtTCTP over-expression (AtTCTP); **b** indicates wildtype (wt) vs. over-expression of AtTCTP-GFP (AtTCTP-GFP). Click here for file
