# Supplementary figures and images for: The effect of Translationally Controlled Tumour Protein (TCTP) on programmed cell death in plants
Source: BMC Plant Biol. 2013 Sep 16;13:135. doi: 10.1186/1471-2229-13-135 (PMC3847524; doi:10.1186/1471-2229-13-135)

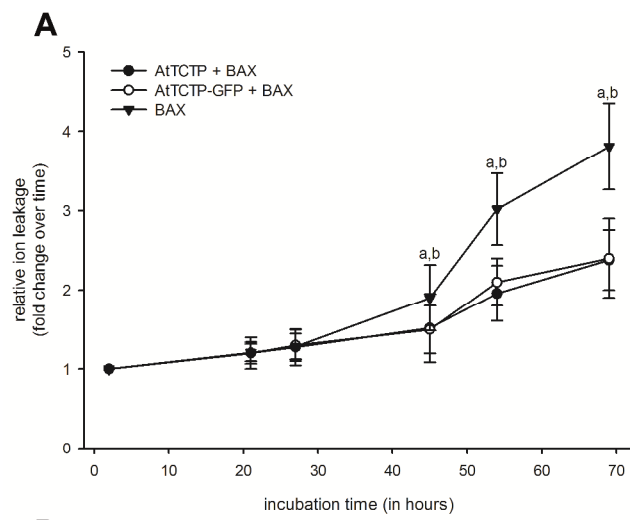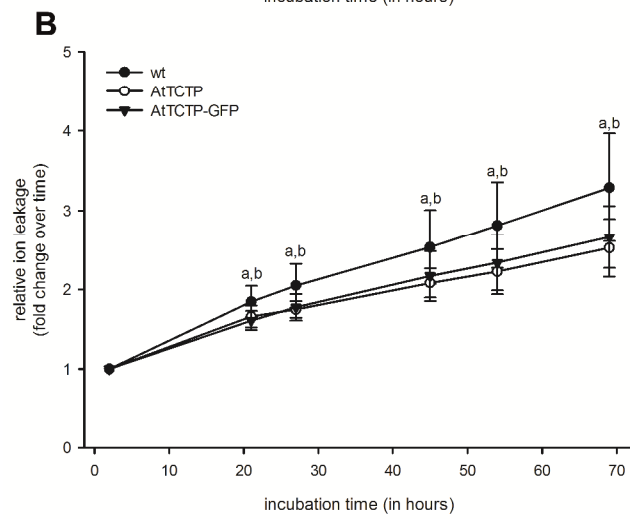

Supplement: Additional file 1: Figure S1 — Effect of AtTCTP-GFP on cell death induced by BAX or tunicamycin. Description of data: Confirmation of AtTCTP-GFP functionality in tunicamycin and BAX experiments. Cell death quantification of tobacco leaf discs transiently over-expressing either AtTCTP or AtTCTP-GFP. A and B show ion leakage calculated relative to time point T0 in response to either expression of BAX induced by 2 μM dexamethasone (DEX) (A), or in response to 2.5 μg · ml-1 tunicamycin (B). Statistical significances were calculated using analysis of variance (P < 0.05) and are indicated as follows: for A: a indicates BAX over-expression (BAX) vs. over-expression of both BAX and AtTCTP (AtTCTP + BAX), b indicates BAX over-expression (BAX) vs. over-expression of both BAX and AtTCTP-GFP (AtTCTP-GFP + BAX); for B: a indicates wildtype (wt) vs. AtTCTP over-expression (AtTCTP); b indicates wildtype (wt) vs. over-expression of AtTCTP-GFP (AtTCTP-GFP). [file 1471-2229-13-135-S1.pdf]
